# The diagnosis of infectious diseases by whole genome next generation sequencing: a new era is opening

**DOI:** 10.3389/fcimb.2014.00025

**Published:** 2014-03-06

**Authors:** Marc Lecuit, Marc Eloit

**Affiliations:** ^1^Biology of Infection Unit, Institut PasteurParis, France; ^2^Inserm U1117Paris, France; ^3^Sorbonne Paris Cité, Institut Imagine, Paris Descartes UniversityParis, France; ^4^Division of Infectious Diseases and Tropical Medicine, Necker-Enfants Malades University HospitalParis, France; ^5^Laboratory of Pathogen Discovery, Department of Virology, Institut PasteurParis, France; ^6^PathoQuestParis, France

**Keywords:** next-generation sequencing, infectious disease medicine, microbiology, diagnostic tests, routine, virology, bacteriology

As in other medical fields, the availability of next generation sequencing (NGS) techniques is about to revolutionize diagnostics of infectious diseases. The demonstration of the microbial origin of diseases and their diagnosis were initially based on the demonstration of the presence of a given pathogen in a given clinical sample, and was first dominated by culture assay for bacteria and later for viruses. These techniques do not advance prior hypotheses regarding the causative agents except their cultivability. In order to seek specific pathogens, specialized media—rich or selective—and culture conditions—defined oxygen tension or temperature—can be used. These techniques suffer a number of limitations, including the need for a dedicated specialized staff and their intrinsic inefficiency in the propagation of fastidious bacteria and several major viruses (*Treponema palidum*, *Mycobacterium leprae*, Hepatitis A, B, C and E viruses). They have been progressively complemented and sometimes replaced by nucleic acid-based tests like PCR or NASBA. The advantages of PCR are numerous: speed, low cost, automation, sensitivity, and specificity. The main drawback of targeted, pathogen-specific PCR is that it is only able to identify predefined targets, which supposes that the physician has elaborated an etiological hypothesis. Moreover, for a series of pathogens, and in particular highly variable RNA viruses like enteroviruses or DNA viruses such as papillomaviruses and adenoviruses that comprise multiple types, PCR-based tests target conserved loci that do not discriminate between genotypes.

To bypass these difficulties, several strategies have been developed, all of whose main objective is to broaden the range of detection. Direct hybridization of non-amplified or random amplified nucleic acids (NA) from samples on DNA arrays has not been proven satisfactory, mostly owing to its relative lack of sensitivity for medical diagnosis. Bacterial typing can be achieved by sequencing the 16S gene or other regions of the genome that are sufficiently conserved to allow definition of consensus primers yet sufficiently variable to allow for typing. Use of NGS has increased the depth of sequencing by several orders of magnitude and thereby the capacity to detect rare species. Nevertheless, with 16S PCR, the taxonomic assignation remains often at the level of the genus, an intrinsic limit due to the conservation of the locus between species of the same genus.

Multiplexed PCR assays for multiple loci have been and are still being developed to provide, at least in principle, simultaneous detection of several agents. Amplicons of multiplexed PCRs can be detected by multiple labeled probes. For example, LightCycler SeptiFast (LC-SF) is a real-time multiplex PCR test able to detect 25 common pathogens responsible for bloodstream infections. A meta-analysis of 34 studies enrolling 6012 patients with suspected sepsis demonstrated an overall sensitivity and specificity of 0.75 (95% CI: 0.65–0.83) and 0.92 (95%CI: 0.90–0.95), respectively, to detect bacteremia or fungemia (Chang et al., 2013). Some multiplex PCR assays can be restricted to certain syndromes to limit the range of pathogens to be tested simultaneously, such as, for example, respiratory infections (Dabisch-Ruthe et al., 2012). The range of multiplex PCR can be considerably improved by designing primers targeting numerous pathogens and varied loci within pathogens and resolving these amplicons using electrospray ionization-mass spectrometry (Wolk et al., 2012) or NGS (Arena et al., 2014). Nevertheless, detection by ionization-mass spectrometry is not based on the determination of the sequence of the amplicon, in contrast to NGS. Diagnostic kits targeting nosocomial pathogens or influenza virus are available. It remains to be seen, however, whether such highly multiplexed PCRs can be applied to a wide range of pathogens, some of which are highly variable in sequence, without losing the analytical sensitivity of single PCR, one of the major advantages of the technique. Moreover, the design of numerous primers will have to be constantly updated along with increase in the number of sequences in databases and identification of new pathogens, in order to maintain a high range of detection. Indeed, addition of a new primer pair to an already highly multiplexed PCR requires some degree of revalidation, which can become a laborious and never-ending process.

An alternative strategy takes advantage of the increasing availability and speed and decreasing cost per base of NGS offered by deep sequencing machines. It is now possible to use the tools of metagenomics, which is the study of the microbial genetic sequences recovered directly from a given human, animal, or environmental sample. In this setting, the sequence of all the NA species of the sample are determined and compared with those in databases. This technology has first been used to describe the complexity and the dynamics of microbiomes from different origins, including from the gut, other mucosal sites and the skin, as well as from various human-made (e.g., sewage) and natural (e.g., sea) environments. It has also been used to discover new infectious agents. De novo assembly of full length genomes of pathogens can sometimes be achieved directly from the samples, and if not large partial sequences can be subsequently completed by using classical molecular biology tools. Frequently, such metagenomic study uncover known but unexpected viruses, phages, bacteria, parasites or fungi (De Vlaminck et al., 2013), which paves the way to application in the field of diagnosis of infectious diseases. As reviewed recently (Barzon et al., 2013; Capobianchi et al., 2013), some applications for NGS in virology—pathogen discovery, study of viral variability—have already emerged.

In principle, such a whole genome NGS (WG-NGS) would be advantageous in clinical diagnostics, as there is no need to design specific primers to pre-amplify target sequences. This avoids the very hard work consisting of designing several tens or hundreds of specific primers able to target multiple pathogens, and checking their capacity to function simultaneously without interference. Furthermore, there is no requirement for continuous adaptation of the sequence of primers with the description of new variants and species.

These advantages, however, come with several drawbacks. The main one is that random amplification, currently indispensable for all available sequencing technologies, also amplifies host NA, meaning that searching for microbial NA is like looking for a needle in a haystack. Indeed, while the depth of sequencing can compensate, at least in part, for this shortcoming, it is not cost-effective. The microbe vs. host NA ratio must therefore be increased using different strategies, such as hydrolysis, chemical treatment or depletion of host sequences. Nevertheless, this procedure still requires high depth sequencing, at least if an analytical sensitivity similar to that of diagnostic PCRs is expected. Also, good genome coverage is necessary to predict phenotypes such as resistance to antimicrobials or virulence, as loci of interest are not specifically targeted and success in obtaining the necessary genetic information is unpredictable when partial sequences are acquired.

The analytical sensitivity of WG-NGS is not as easy to evaluate as that of PCR, as it is more critically influenced by matrix properties. In particular, the quantity of host NA, as well as its physical state or association with proteins, may complicate its elimination before sequencing. Also, the analytical sensitivity critically depends on the depth of sequencing. Using around 20,000–100,000 reads of the 454 platform per sample, only a high load of the Schmallenberg virus (superior to 10^10^ gc/mL) could be detected in clinical samples (Rosseel et al., 2012). Increasing the depth of sequencing for an optimized sample preparation can decrease the level of detection down to 10^2^–10^3^ gc/mL, within the range of most homemade PCRs. Also, in contrast to PCR, the analytical sensitivity depends on the length of the genome. Longer length translates into a higher number of potentially available reads as seen in some studies for viruses (Wylie et al., 2012). This should also be the case for bacterial and fungal genomes, which could be seen as an advantage for the detection of such microbes as their blood concentration can be very low even in samples of infected patients. The diagnostic sensitivity was evaluated in some studies. Sequence analysis of the human virome in febrile and afebrile children revealed a wide range of viruses in plasma that correlated with the febrile status (Wylie et al., 2012). Of note, this study illustrated that compared with PCR, WG-NGS missed some samples found positive with high CT by qPCR, a shortcoming that was partially overcome by increasing the depth of sequencing. That can be partially overcome by improving the sample preparation. Moreover, their work revealed two advantages of NGS-WG: first, viruses were identified that would not have been routinely queried by PCR assays for known pathogens (for example astrovirus MLB2 in plasma). Also WG-NGS enabled determination of virus subtype or variant strains of rhinovirus, bocavirus and HHV-6, even on the basis of a few reads, without sequencing most of the viral genome. Microbial and DNA virus loads in plasma were also followed efficiently after immunosuppressive therapy (De Vlaminck et al., 2013). In the field of bacteriology, most studies have dealt with sequencing of clinical isolates cultured *in vitro*, but good results have been obtained by direct sequencing from clinical samples, for example for the diagnosis of tuberculosis lesions (Chan et al., 2013), fecal samples from diarrheic patients (Loman et al., 2013), or urinary samples from patients with suspected urinary tract infections (Hasman et al., 2014). Another advantage of the technique is its capacity to identify co-infections, which is of great help to adapt therapeutics.

Developing a WG-NGS diagnostic pipeline critically relies on two partly interdependent criteria: time to results and database exhaustiveness. Indeed, some sequence knowledge is necessary to design primers for PCR, but the whole genome sequence does not need to be known. Indeed this is also the case for WG-NGS, but lack of information regarding the whole genome sequence and organization will have an impact on sensitivity (some useful reads being at risk of not being properly identified). As the growth of databases is very rapid, being fueled by the development of NGS as a standard tool, such limits will not last long. Also, the requirements are not the same for pathogen discovery, when the range of detection should typically include the unknown, and medical diagnosis. In this latter case, it is more important to screen samples against a curated database of known pathogens that could be of interest for the physicians. Typical blast analysis of hundreds of million of reads after de novo assembly into larger contigs against the whole NCBI databases using relaxed criteria, which is classical in pathogen discovery, is too time- and resource-consuming to be used in diagnostics. By contrast, stringent mapping of non-assembled reads on a comprehensive database of pathogens, together with the progressive increase of read length permitted by the evolution of sequencers, speeds up the overall process down to a few hours. Time from sample to results can thus be 2 days or even less, which is useful for some indications. Indeed, this time to results still remains much longer than the few hours needed for some PCRs and the needs of critical care (<8 h).

The question is probably not if, but rather when, WG-NGS will become a routine test in diagnostics of infectious diseases. This development will require improvement in sample preparation, availability of sequencers in central laboratories and validated pipelines for read sorting and taxonomic assignation. There is no doubt that such an opportunity will sooner than later profoundly change the routine laboratory practice together with the means of conducting microbiological diagnosis.
